# Safety and Suitability of an Infant Formula Manufactured from Extensively Hydrolysed Protein in Healthy Term Infants

**DOI:** 10.3390/nu15081901

**Published:** 2023-04-14

**Authors:** Lindsey Otten, Elisabeth Schelker, Hanna Petersen, Antonia Nomayo, Manja Fleddermann, Bianca M. Arendt, Theresa Britzl, Elisabeth M. Haberl, Frank Jochum

**Affiliations:** 1 Department of Pediatrics, Evangelisches Waldkrankenhaus Spandau, Stadtrandstr. 555, 13589 Berlin, Germany; lindsey.otten@jsd.de (L.O.); elisabeth.schelker@jsd.de (E.S.); hanna.petersen@jsd.de (H.P.); antonia.nomayo@jsd.de (A.N.); 2 HiPP GmbH & Co. Vertrieb KG, Georg-Hipp-Str. 7, 85276 Pfaffenhofen an der Ilm, Germany; manja.fleddermann@hipp.de (M.F.); bianca.arendt@hipp.de (B.M.A.); theresa.britzl@hipp.de (T.B.); elisabeth.haberl@hipp.de (E.M.H.); 3 Brandenburg Medical School Theodor Fontane (MHB), Fehrbelliner Str. 38, 16816 Neuruppin, Germany

**Keywords:** extensively hydrolysed whey protein, protein hydrolysate, infant formula, infant nutrition, infant weight, infant growth, breastfed, neonatal

## Abstract

We aimed to demonstrate that healthy term infants experience noninferior growth with infant formula manufactured from extensively hydrolysed whey protein (eHF) compared to intact cow’s milk protein (control formula, CF). This prospective, randomised, double-blind, parallel-group, controlled, multicentre trial included healthy term infants who were exclusively formula-fed. Infants ≤ 25 days of age received eHF or CF for at least three months up to 120 days of age, with a follow-up until 180 days of age. A reference group included exclusively breastfed infants (BF). Of 318 infants randomised, 297 (148 CF, 149 eHF) completed the study per protocol. Weight gain up to 120 days of age was noninferior (margin −3.0 g/day) in eHF (28.95 (95% CI: 27.21; 30.68) g/day) compared to CF (28.85 (95% CI: 27.10; 30.61) g/day) with a difference in means of 0.09 g/day and a lower limit of the one-sided 97.5% CI of −0.86 g/day (*p* < 0.0001 for noninferiority testing). Weight gain remained comparable during follow-up. Further anthropometric parameters did not differ between the infant formula groups throughout the study. Growth was comparable in BF. No relevant safety issues were observed. To conclude, eHF meets infant requirements for adequate growth during the first six months of life and can be considered safe and suitable.

## 1. Introduction

Nutrition during infancy plays a critical role in the development of infants and is linked to long-term health effects [1]. Human milk is the optimal source of nutrition for infants, containing all essential nutrients required for growth and development during the first 4–6 months of life [2,3,4]. In addition, there is clear evidence for major health benefits of breastfeeding for both the infant, for example through lower rates of infections, type 1 and type 2 diabetes and the metabolic syndrome, or protective effects against obesity [5,6,7,8,9], and the mother, such as reducing the risk for breast cancer [9,10] and type 2 diabetes [11]. However, infants who do not (exclusively) receive human milk are entitled to the best alternatives possible [12]. Therefore, different infant formula types are available to best meet the needs of these infants. Infant formulae manufactured from partially or extensively hydrolysed protein, marketed as HA (Previously marketed as hypoallergenic formula) formula, have been recommended or suggested as possible alternatives to intact cow’s milk protein-based formulae for infants who are not exclusively breastfed and have a hereditary risk of developing atopic diseases [13,14,15,16].

Several studies and systematic reviews have demonstrated normal growth in term [15,17,18] as well as in preterm infants who are fed formulae manufactured from protein hydrolysates [19,20,21]. Protein hydrolysates, as a source of protein in infant and follow-on formulae, have been allowed under Directive 2006/141/EC [22] for many years. Del. Reg. (EU) 2016/127 now requires the safety and suitability of each specific protein hydrolysate to be established by clinical evaluation [23].

The present study was specifically designed to evaluate the safety and suitability of an infant formula manufactured from extensively hydrolysed whey protein (eHF, intervention formula) compared to an infant formula manufactured from intact cow’s milk protein (control formula, CF), following guidelines from the European Food Safety Authority (EFSA) [24,25]. The noninferiority of eHF compared to CF was assessed with respect to mean daily weight gain, as a recognised safety parameter for infants, with a noninferiority margin of −3.0 g/day [26].

## 2. Materials and Methods

### 2.1. Study Design and Population

The “HA Safety in Infants” (HASI) study was a prospective, randomised, double-blind, parallel-group, controlled, multicentre, noninferiority trial. Twenty-one study sites in Europe (11 in Bulgaria, one in Germany, eight in Hungary, and one in the Czech Republic) participated in the study from March to October 2021. The study was conducted in accordance with ICH Good Clinical Practice and the Declaration of Helsinki, as far as they are applicable to an infant formula study, and in accordance with the local legal and regulatory requirements. The study was approved by the corresponding ethics committees and registered at ClinicalTrials.gov (NCT04736082).

The main interventional study period lasted a minimum of three months from enrolment (≤25 days of age) until 120 days of age, with a subsequent voluntary follow-up until 180 days of age. Exclusive infant formula feeding began no later than 26 days of age. After the enrolment visit (V0, 0–25 days of age), infants attended study visits at 30 ± 3 (V1), 60 ± 3 (V2), 90 ± 3 (V3), 120 ± 3 (V4) and 180 ± 7 (follow-up, V5) days of age. After 120 days of age, complementary foods/drinks could be introduced, as recommended.

Healthy, term infants up to 25 days of age from singleton pregnancies and with a gestational age between ≥37 weeks + 0 days and ≤41 weeks + 6 days, birth weight between ≥3rd and ≤97th percentile per gestational age [27], as well as the mothers’ intention to exclusively formula feed (eHF/CF group) or breastfeed (BF group) their infants for reasons not related to the study, were eligible for participation. Exclusion criteria included adverse foetal or infant medical history like severe acquired or congenital illness, or chromosomal anomalies (if known) that are expected to interfere with normal feeding or growth, intensive care prior to or at V0, infants under (ongoing) antibiotic treatment longer than three days (72 h) before or at V0, feeding difficulties or infant formula intolerance, or participation in another clinical trial. Furthermore, parental medical history, including parental disease that may have an impact on the study’s conduct or that may have an influence on infant growth and feeding behaviour based on the investigator’s opinion, infants born to mothers with medical conditions that have an effect on the infant’s gastrointestinal tract/ability to be fed and/or growth (e.g., insulin-dependent diabetes mellitus), or recreational drug or alcohol intake by the mother during the last two trimesters of pregnancy led to exclusion.

Upon written informed consent of both parents, eligible infants were enrolled in the study and randomised to one of the two infant formula-fed groups (FF), i.e., eHF or CF. Randomisation was carried out in a double-blinded dynamic manner, stratified by country and sex with a centralised randomisation management interface to achieve allocation concealment.

Infants who met the above criteria and whose mothers intended to exclusively breastfeed for at least three months up to 120 days of age were assigned to the reference group in a nonrandomised manner. Recruitment in the breastfed group (BF) was based on each study site’s intervention recruitment rate at a ratio of 4:1 (FF:BF) in order to achieve a similar distribution of breastfed infants across the study centres and recruitment period compared to the formula-fed infants.

### 2.2. Study Product

The two infant formulae complied with the requirements defined in Art. 3 Del. Regulation (EU) 2016/127 [23] as well as Art. 5 Directive 2006/141/EC [22] and had a similar composition. The protein content of both infant formulae was 1.9 g/100 kcal and the formulae were isocaloric (Table 1). The infant formulae (manufactured by HiPP GmbH & Co. Vertrieb KG, Pfaffenhofen an der Ilm, Germany) were based on different protein sources: eHF was manufactured from extensively hydrolysed whey protein (Peptigen^®^ IF-3080, Arla Foods Ingredients, Videbæk, Denmark), and CF from intact cow’s milk protein.

The infant formulae were packaged in identically designed and labelled boxes. Both consisted of similar yellowish-white, fine-grained powder.

### 2.3. Anthropometric Measurements

Measures of body weight (g), length (cm) and head circumference (cm) were taken at each visit at the study site. All measurements were repeated and recorded twice. Infants were weighed naked on a calibrated, medical weighing scale and for each site the same scale was used for all infants at all visits. The recumbent length was measured with a standard measuring table and was recorded to the nearest 0.1 cm. Head circumference was measured using an insertion tape and also recorded to the nearest 0.1 cm.

The study was conducted during the Coronavirus Disease 2019 (COVID-19) pandemic. In case of restricted access to the study sites or safety concerns for the subjects, it was possible to perform home visits by trained study personnel for V1 until V4. If home visits were not feasible, V2 and V3 could be performed at home by the parents under the advice of a study team member via telemedicine.

### 2.4. Infant Formula Intake

Infant formula intake (number of feedings per day, volume of consumed infant formula, intake of other foods and drinks) was documented in 3-day diaries, which were completed by the parents during the three days prior to each visit (starting from V1).

### 2.5. Overall Health and Adverse Events

Illness and medication were documented by parents throughout the study in the Disease and Administration Journal. At each visit, the investigator inquired on and documented symptoms of illness since the previous visit, as well as medications, including the reason for intake, frequency of intake and duration of use.

Adverse and serious adverse events (AEs, SAEs) were documented throughout the study. Investigators followed up on (S)AEs until they were resolved.

### 2.6. Statistical Analysis

The primary study outcome was the mean daily weight gain (g/day) over a period of three months (between 30 days of age (V1) and 120 days of age (V4); calculated by subtracting the weight obtained at V1 from the weight obtained at V4 divided by the number of days passed between those visits). Secondary outcomes included daily weight gain between V1 and the other visits (V2 to V5), as well as daily gain for additional anthropometric measures (body length, head circumference, body mass index (BMI)), anthropometric z-scores compared with the WHO 2006 Child Growth Standards z-scores [28]; average daily intake of the study product (amount, energy, macronutrients) and other food and drinks; the number of feedings; and AEs categorised by System Organ Class and by Preferred Term using the Medical Dictionary for Regulatory Activities (MedDRA^®^; version 24.1) (The MedDRA^®^ terminology is the international medical terminology developed under the auspices of the International Council for Harmonisation of Technical Requirements for Pharmaceuticals for Human Use (ICH). MedDRA® trademark is registered by ICH).

A linear mixed model was applied to assess the primary outcome (comparison of eHF and CF for mean daily weight gain between V1 and V4) and included infant formula group and sex (randomisation stratification factor) as fixed factors, centre as a random factor, and weight at V1 (baseline) as a covariate. Noninferiority of eHF was shown if the lower limit of the one-sided 97.5% CI for the difference of the mean daily weight gain between eHF and CF was greater than −3.0 g/day [26]. The *p*-value of noninferiority was expressed with regard to the predefined margin of noninferiority. The adjusted means (least squares means) were calculated in each group considering the observed proportions and means for covariates.

Secondary endpoints, including daily gain in weight between V1 and each visit (V2 to V5) and in further anthropometric parameters were analysed independently using the same model as for the primary outcome. As a sensitivity analysis, daily gains for anthropometric parameters were analysed using linear mixed models for repeated measurements at each visit (including infant formula group, the visit, the interaction between the group and the visit, baseline value at V1 and sex as fixed factors, and the subject and centre as random factors). Absolute values of anthropometric measures, anthropometric z-scores and infant formula intake at each visit were analysed using linear mixed models for repeated measurements.

Inferential tests focused on an explorative two-sided 5% significance level, except for noninferiority tests of mean daily weight gain, which used a one-sided 2.5% significance level.

All analyses were conducted in both the full analysis set (FAS) and the per-protocol set (PPS). In both sets, only infants who met the eligibility criteria were included. The FAS was defined as all infants (all infants in BF, randomised infants in FF) who participated in V1 and received the study product at least once (if in FF). The per-protocol set (PPS) was defined as all infants from FAS without major deviations to the protocol, including visits outside the visit window, specified medications or complementary food and drink up to V4. Since this is a noninferiority study, the main analysis was performed for the PPS. Results for the primary outcome are also shown for FAS as sensitivity analyses. Two other sensitivity analyses of the primary outcome consisted of an unadjusted analysis (data not shown) and an analysis with adjustment on possible confounding factors (birth weight, gestational age, age of mother at infant birth, BMI of mother at delivery and educational level of mother). The safety analysis is presented for the intention-to-treat population (ITT), i.e., all infants randomised in the FF groups and all infants included in the BF group.

Based on previous observations [29,30,31], infant weight gain was estimated to be ~29 g/d with a standard deviation (SD) of ~7 g. A one-sided, two-sample *t* test procedure to show noninferiority and assuming a true difference of 0.0 g in mean daily weight gain (pooled SD of 7.0 g) with a noninferiority margin of −3.0 g/day (type-one error of 2.5%, power of 80%) resulted in 87 necessary study participants per group. Assuming a drop-out rate (including major protocol deviators) of 40%, it was estimated that 145 infants per FF group (excluding screening failures) were needed at V1 (equal allocation to both study groups). To compensate for the potential early termination of infants between enrolment and V1, an additional 30 infants were enrolled. Furthermore, up to 40 infants were included in the nonrandomised BF reference group (not based on sample size estimation). The BF group was not included in statistical analyses.

Descriptive analyses were performed by group and by visit for all parameters. Mean (SD) or *n* (%) are presented.

Statistical analyses were performed using the software SAS^®^ version 9.4 (SAS Institute Inc., Cary, NC, USA). The software R version 4.1.0 (May 2021) [32] was used for some visualisations and graphs and for the calculation of anthropometric z-scores compared with the WHO 2006 Child Growth Standards (package ‘anthro’ version 0.9.4) [33].

### 2.7. Compliance

The study product should be the only source of nutrition for FF infants, meaning no breastfeeding, no complementary food, no energy-containing liquids, no feedings of any other infant formula and no energy-free drinks like water or unsweetened tea should be consumed until 120 days of age. Infants who received any of these liquids or complementary foods before 120 days were not included in the PPS analysis.

## 3. Results

### 3.1. Study Participants

**Figure 1:**
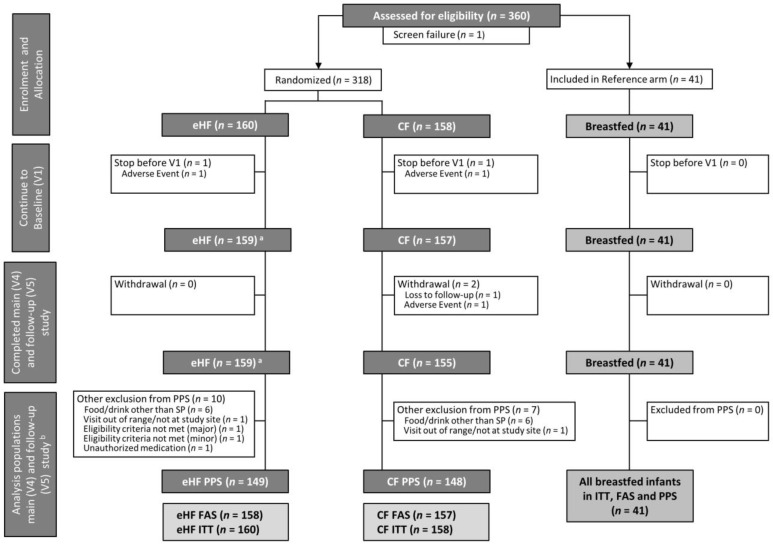
Flow chart of subject enrolment and distribution into formula-fed groups and breastfed reference group, ^a^ One infant of which could not be analysed within the FAS due to eligibility criteria not met. ^b^ All infants who completed V4 also completed V5. The analysis populations (ITT, FAS, PPS) for main study (V4) and follow-up (V5) are identical. CF: control formula; eHF: infant formula manufactured from extensively hydrolysed whey protein; FAS: full analysis set; ITT: intention-to-treat population; SP: study product; PPS: per protocol set; V: visit.

In total, 318 infants were randomised to FF, 41 breastfed infants were included in BF (Figure 1). The drop-out rate was low and overall compliance was high. Twenty-one participants from FF had major protocol deviations or did not complete the main study up to V4. All participants who completed V4 also completed V5. There was no apparent difference between eHF and CF regarding the frequency of deviations or proportion of infants that were excluded from PPS. All infants in BF completed the study per protocol.

Most study participants were enrolled in Bulgaria (94%), followed by Hungary (6%). The proportion of participants recruited in Germany and the Czech Republic was less than 1%. All breastfed infants were enrolled in Bulgaria.

### 3.2. Study Population Characteristics

The main socioeconomic and other baseline characteristics of FAS are outlined in Table 2. There were no apparent differences in birth or parental characteristics or in anthropometric parameters at birth between eHF and CF (Table 2). BF was similar to FF, except for sex and mode of birth. In BF, there were more females, and the majority of infants were born vaginally compared to FF (Table 2).

### 3.3. Primary Outcome: Weight Gain up to V4

In PPS, the adjusted mean [95% CI] of weight gain between V1 and V4 was 28.95 [27.21; 30.68] g/day and 28.85 [27.10; 30.61] g/day in eHF and CF, respectively, corresponding to a difference in means of 0.09 g/day.

**Figure 2:**
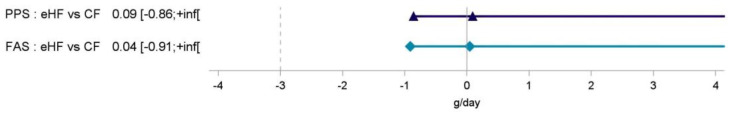
Difference in mean weight gain (g/day) with one-sided 97.5% interval between infant formula feeding groups from 30 days of age (V1) to 120 days of age (V4). CF: control formula; eHF: infant formula manufactured from extensively hydrolysed whey protein; FAS: full analysis set; inf: infinity; PPS: per protocol set; V: visit.

Noninferiority of eHF compared to CF was shown in PPS and FAS by confirming that the lower limit of the one-sided 97.5% CI for the difference in mean daily weight gain, (−0.86 g/day in PPS, −0.91 in FAS) was greater than −3.0 g/day (*p* < 0.0001 for noninferiority testing) (Figure 2).

Significant associations of early weight gain with the covariates infant baseline weight, gestational age, sex, and mother’s BMI at delivery, depending on the analysis population, were detected when potential confounding factors were included in a sensitivity analysis (Table S1).

### 3.4. Secondary Parameters: Further Growth Parameters

Infant weight, length and head circumference increased consistently throughout the study, with adequate growth in all groups. Independent of the statistical model used, there were no statistically significant differences between FF groups in absolute values of weight, length or head circumference at each visit (Table 3) or in gains for each of these parameters between V1 and any subsequent visit (Table S2). Results for FAS and PPS (data not shown) were comparable.

Descriptive statistics on BF infants indicated lower mean daily weight gain compared to FF initially at V2 and V3 and comparable values by the end of the main study period (V1 to V4 (FAS): 29.2 (4.1) g/day) and follow-up (V1 to V5 (FAS): 23.1 (3.8) g/day) (Table S2). As stated above, all infants in BF completed the study per protocol; therefore, FAS and PPS results are identical for BF.

**Figure 3:**
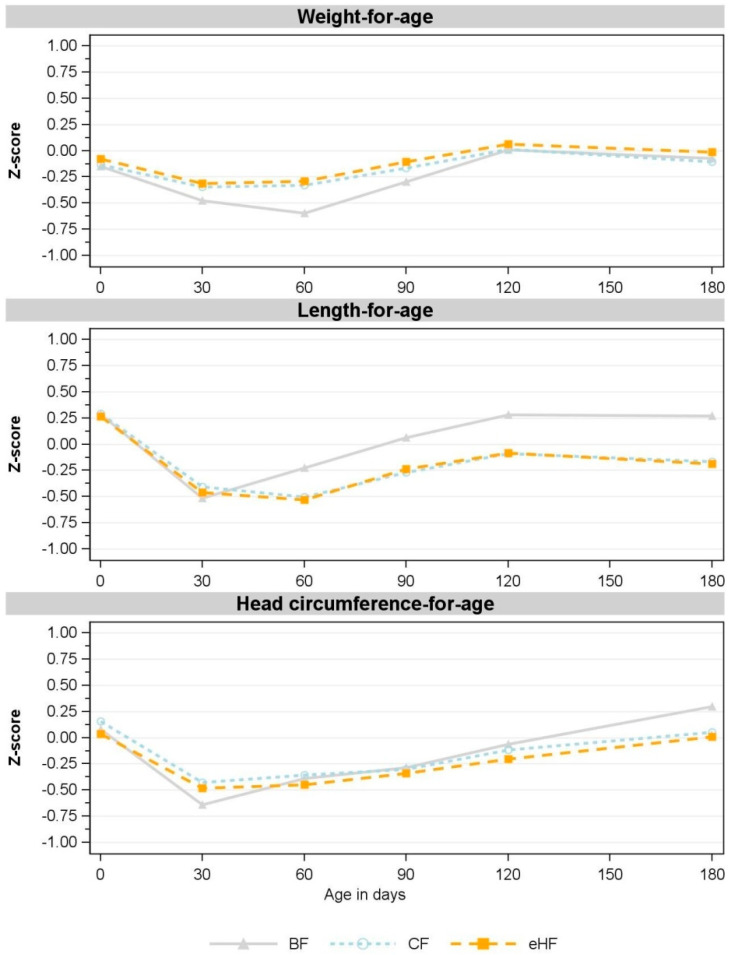
Observed mean for weight-for-age, length-for-age and head circumference-for-age z-scores between birth and 180 days of age (V5) (FAS). V1: 30 days; V2: 60 days; V3: 90 days; V4: 120 days; V5: 180 days. Missing values in CF: weight-for-age: V1: *n* = 1; V3: *n* = 3; V4, V5: *n* = 2; length-for-age: V1, V3: *n* = 1, V4, V5: *n* = 2; head circumference-for-age: V1: *n* = 1, V3, V4, V5: *n* = 2. Missing values in eHF: length-for-age: V4: *n* = 1. Missing value in BF: head circumference-for-age: V3: *n* = 1. BF: breastfed reference group; CF: control formula; eHF: infant formula manufactured from extensively hydrolysed whey protein; FAS: full analysis set; n: number of observations; V: visit.

Z-scores derived using WHO Child Growth Standards [28] were comparable between eHF and CF and indicated no statistical differences at any time point (Figure 3). All mean z-score values were within ±0.75.

Consistent with the absolute weight and absolute length, corresponding z-scores for FF deviated slightly from those of the BF group. FF had higher weight-for-age z-scores from V1 to V3, while length-for-age z-scores were lower from V2 to V5 compared to BF (Figure 3). Both FF groups also had higher BMI-for-age and weight-for-length z-scores than BF at all visits (Figure S1). Head circumference-for-age z-scores were comparable in all groups throughout the study (Figure 3).

### 3.5. Infant Formula Intake

Most infants were compliant with the feeding instructions. Seven infants in eHF and nine infants in CF were excluded from PPS due to major deviations in feeding instructions; most of these were the consumption of low amounts of drinks without energy (water or unsweetened tea). No complementary foods were given before V4. All 41 infants in the BF group were fully compliant with the feeding instructions, i.e., exclusively breastfed until V4. Between V4 and V5, only a few infants received complementary foods or drinks. At V5, the proportion of energy from the study product (mean (SD)) was 97.3 (9.1) % in eHF and 97.8 (8.4) % in CF.

The average number of infant formula feedings per day and mean daily amount of infant formula (average over three days) were largely comparable in the FF groups throughout the study in FAS (Table 4) and also in PPS (Table S3). At V3, infants from FAS in eHF consumed significantly more infant formula than infants in CF (Table 4). The same was observed in PPS (Table S3). While the average daily number of feedings decreased from V1 to V5, the average daily amount of infant formula increased consistently.

BF infants received a comparable number of feedings per day as FF infants (Table 4 and Table S3).

### 3.6. Adverse Events Assessment

Based on the low frequency of AEs, no inferential statistics were performed. A total of 17 AEs in 16 infants were reported up to V5 in the ITT population (Table 5); all AEs were intervention emergent, i.e., starting after enrolment. In the FAS, 14 AEs occurred in 13 infants (six AEs in five infants (3.2%) in CF, eight AEs in eight infants (5.1%) in eHF). Comprehensive safety documentation and continuous safety monitoring showed no clinically relevant differences regarding the incidence and overall occurrence of AEs between the FF groups.

A summary of the AEs in the ITT by MedDRA System Organ Class and Preferred Term are provided in the Supplementary Material (Table S4).

Most AEs were of mild intensity. Only three AEs, which were all of moderate intensity, were linked to the intake of one of the study products. These three product-related AEs resulted in the stop of study product intake, withdrawal of the corresponding infants from the study and withdrawal of the informed consent. Two of the infants were prematurely withdrawn before V1 (one CF, one eHF) and were therefore excluded from the FAS. Reported symptoms were fussing and regurgitation (CF), as well as crying and fussing (eHF). One additional infant with an AE related to the study product remained in the FAS (CF), since study participation was stopped after V2. The reported symptoms were crying, fussing and rash on face and body.

All AEs were resolved (recovery without sequelae) at the end of the study (FAS). No AEs were reported in BF.

Only one SAE was reported concerning one infant in the CF group with hospitalisation due to a diagnosed urinary tract infection. The SAE was not linked to study product intake or the research and was resolved to full recovery.

### 3.7. Influence of the Coronavirus Pandemic

Although the trial was conducted during the Coronavirus Disease 2019 (COVID-19) pandemic, most visits could be conducted at the study sites. The number of visits conducted at home by study personnel or via telemedicine was very low (FAS: 0.49%).

## 4. Discussion

In this randomised controlled study, healthy term infants fed eHF compared to CF experienced noninferior weight gain during the first 180 days of age. Moreover, further growth parameters, including absolute weight, length and head circumference as well as z-scores, did not differ significantly between the two FF groups and were within ranges for normal growth [28]. With regard to the frequency and type of AEs, no safety concerns arose.

The observed weight gain in both FF groups was similar to results reported in other studies using the same protein source of extensively hydrolysed whey protein (Peptigen^®^ IF-3080) [29] and the same whey:casein ratio using intact cow’s milk protein [30,31]. Furthermore, weight gain during the period of at least three months of exclusive formula feeding was comparable to recent literature on infant formula manufactured from intact or hydrolysed (partially or extensively) protein ranging between 28.0 and 31.4 g/day [18,29,30,31,34]. The noninferiority of infant formula manufactured from hydrolysed whey protein was in line with results reported in other publications. Two randomised controlled trials [17,18] and one pooled analysis [35] demonstrated comparable growth in infants fed infant formula manufactured from partially hydrolysed whey protein compared to intact cow’s milk protein during the first four to five months of age.

Analysis of covariates indicated that baseline weight, as well as sex, gestational age and maternal BMI had a significant effect on weight gain between V1 and V4. Literature confirms that weight at birth affects infant growth during the first months of life [36,37,38]. Also, the impact of gestational age [39] and maternal BMI [40] on infant weight is well documented. Furthermore, sex difference during early growth is well documented in the scientific literature, and thus was included as a stratification factor during randomisation. Different reference growth charts are published for girls and boys [28,41], showing that weight, length and head circumference gain is lower in girls than in boys. The adjustment of baseline covariates (which corresponds to weight at baseline/V1) is required by authorities in clinical trials to correct for systematic differences at baseline between groups [42,43] and was considered for all statistical models in the presented study.

The decrease in weight-for-age, length-for-age and head circumference-for-age z-scores between birth and V1, which was observed in all groups, may be explained by the standardisation of measurements in our study from V1 on, while measurements at birth were performed at maternity wards by non-study personnel.

Concerning nutritional intake, infants in the two FF groups had comparable intake of study product based on the number of feedings, daily amounts and energy intake throughout the study. The slightly and significantly higher mean daily intake in eHF compared to CF at V3 of approximately 18 mL/day is considered clinically irrelevant. The total amount of study product intake in both FF groups (with a mean ranging from 738 to 858 mL/day) throughout the study as well as the small difference in intake between CF and eHF observed at V3 was in line with results described in other studies, where intakes between 618 and 1094 mL/day were reported for the period of one to four months of age [18,29,44]. The mean total daily energy intake (mean from 487 to 566 kcal/day; almost exclusively from study product) observed throughout the study was in accordance with values published by EFSA (449 to 546 kcal/day) for infants between one and five months of age [45].

No statistical comparison to the nonrandomised BF reference group was performed; therefore, the analysis is purely descriptive. Despite some fluctuations throughout the study in, for example, weight-for-age and length-for-age z-scores in BF, values remained well within the normal range. BF infants in the present study experienced similar growth rates as FF infants by the end of the main study period (V4) and through to V5. Specifically, mean daily weight, length and head circumference gain from V1 to V5, as well as weight-for-age z-scores at V5, were comparable between BF and FF infants. Although lower weight gain in BF compared to FF infants has been frequently observed [18,29,30,46], similar weight gain between the groups has also been reported [47,48].

The study products used in the present study have an energy content (66 kcal/100 mL) that is close to mature human milk and a slightly reduced protein content of 1.2–1.3 g/100 mL (1.9 g/100 kcal) in comparison to standard infant formula [49]. Furthermore, a recent meta-analysis reports breastmilk intake in exclusively breastfed, healthy term infants of 735 to 729 mL/day at three and six months, respectively [50]. Together with the similar feeding frequency, this may explain the observed similar growth between FF and BF in the present study. Scientific literature on infant formula studies indicates that higher protein intake in infancy can increase the risk of early weight gain and childhood overweight and obesity [1,51].

Overall, our study had a low dropout rate and the participants were highly compliant, particularly regarding the nutrition instructions. Most infants received only the study product (FF) or were exclusively breastfed (BF) up to 180 days of age. The study products were comparable in terms of energy content, and the energy intake was similar between the FF groups throughout the study, so the protein source was the main difference in the diet between eHF and CF.

Although four European countries were involved in the study, nearly all participants (94%) were recruited in Bulgaria. We do not expect this to affect the generalisability of the results. Although on-demand versus structured feeding practices may differ among countries, the main variation in feeding practices occurs during the introduction of complementary foods, which was not relevant in the present study. Further strengths include the extended observation time up to 180 days of age as well as the inclusion of a breastfed reference group for qualitative comparison.

## 5. Conclusions

The infant formula manufactured from extensively hydrolysed whey protein used in this study met infant requirements for adequate growth compared to the control infant formula manufactured from intact cow’s milk protein and a reference group of breastfed infants. Based on these results, it can be concluded that the extensively hydrolysed whey protein-based infant formula is suitable and safe as the only source of nutrition for infants during the first months of life.

## Figures and Tables

**Table 1:** Macronutrient composition of study products.

Nutrients	eHF		CF	
	Per 100 kcal	Per 100 mL Ready to Drink ^a^	Per 100 kcal	Per 100 mL Ready to Drink ^a^
Energy (kJ)		275		276
(kcal)	100	66	100	66
Protein (g)	1.9	1.2	1.9	1.3
Carbohydrates (g)	10.8	7.1	10.6	7.0
Fat (g)	5.4	3.5	5.4	3.6

^a^ Standard solution as specified by the manufacturer. These are theoretical values derived from calculation based on the recipe. The analysis values of products made of natural raw materials are subject to accepted variations. Both products contained the probiotic strain Limosilactobacillus fermentum CECT5716 (1.0 × 10^7^ CFU/g powdered infant formula at the time of production) and the prebiotic Galactooligosaccharide (0.3 g/100 mL). CF: control formula; eHF: infant formula manufactured from extensively hydrolysed whey protein (Peptigen^®^ IF-3080).

**Table 2:** Birth characteristics, demographic and social factors (FAS).

Variable	eHF (*n* = 158)	CF (*n* = 157)	BF (*n* = 41)
Sex, female (*n* (%))	69 (43.7)	69 (43.9)	23 (56.1)
Gestational age (weeks, mean (SD))	38.8 (1.0)	39.0 (1.0)	38.6 (1.1)
Mode of birth			
Caesarian section, *n* (%)	92 (58.2)	92 (58.6)	17 (41.5)
Vaginal birth, *n* (%)	66 (41.8)	65 (41.4)	24 (58.5)
Weight at birth (g, mean (SD))	3275 (389)	3251 (424)	3233 (452)
Length at birth (cm, mean (SD))	50.0 (1.6)	49.9 (1.7)	50.0 (2.0)
Head circumference at birth (cm, mean (SD))	34.3 (1.2)	34.4 (1.2)	34.2 (1.4)
Maternal age at delivery (years, mean (SD))	28.1 (5.6)	27.4 (5.4)	27.9 (5.7)
Maternal BMI before pregnancy (kg/m^2^, mean (SD))	21.7 (2.7)	21.7 (4.0)	21.2 (2.1)
Maternal weight gain during pregnancy (kg, mean (SD))	15.9 (6.6)	14.7 (5.2)	15.3 (5.3)
Level of education mother			
Primary, *n* (%)	27 (17.1)	36 (22.9)	6 (14.6)
Secondary, *n* (%)	92 (58.2)	83 (52.9)	26 (63.4)
Tertiary, *n* (%)	39 (24.7)	38 (24.2)	9 (22.0)

BF: breastfed reference group; BMI: body mass index (kg/m^2^); CF: control formula; eHF: infant formula manufactured from extensively hydrolysed whey protein; FAS: full analysis set; *n*: number of observations; SD: standard deviation.

**Table 3:** Absolute weight, length and head circumference from 30 days of age (V1) to 180 days of age (V5) (FAS), mean (SD).

Variable	Visit	Age at Visit (Days)	eHF (*n* = 158) ^a^	CF (*n* = 157) ^b^	BF (*n* = 41) ^c^
Weight (g)	1	30	4169 (444)	4155 (500)	4047 (497)
	2	60	5178 (493)	5155 (530)	4928 (518)
	3	90	6058 (550)	6011 (530)	5855 (533)
	4	120	6791 (586)	6744 (536)	6675 (570)
	5	180	7650 (747)	7551 (605)	7509 (635)
Length (cm)	1	30	53.3 (2.0)	53.4 (2.1)	53.1 (2.4)
	2	60	56.7 (2.4)	56.7 (2.5)	57.1 (2.7)
	3	90	60.1 (3.0)	60.1 (3.0)	60.5 (3.3)
	4	120	62.8 (3.1)	62.8 (3.3)	63.4 (3.5)
	5	180	66.3 (3.8)	66.3 (3.9)	67.0 (4.6)
Head circumference (cm)	1	30	36.4 (1.3)	36.4 (1.3)	36.1 (1.5)
	2	60	38.2 (1.4)	38.3 (1.4)	38.1 (1.3)
	3	90	39.6 (1.4)	39.7 (1.4)	39.6 (1.2)
	4	120	40.9 (1.4)	41.0 (1.4)	40.9 (1.3)
	5	180	42.8 (1.5)	42.8 (1.6)	43.0 (1.4)

^a^ Missing values in eHF: length: V4: *n* = 1. ^b^ Missing values in CF: weight: V1: *n* = 1; V3: *n* = 3; V4, V5: *n* = 2; length: V1: *n* = 1; V3: *n* = 1, V4, V5: *n* = 2; head circumference: V1: *n* = 1, V3, V4, V5: *n* = 2. ^c^ Missing value in BF: head circumference: V3: *n* = 1. BF: breastfed reference group; CF: control formula; eHF: infant formula manufactured from extensively hydrolysed whey protein; FAS: full analysis set; n: number of observations; SD: standard deviation; V: visit.

**Table 4:** Feedings characteristics (FAS), mean (SD).

	Visit	Age at Visit (Days)	eHF (*n* = 158)	CF (*n* = 157) ^a^	BF (*n* = 41)
Average number of feedings per day ^b^	1	30	7.1 (0.9)	7.0 (0.8)	7.4 (0.6)
	2	60	6.1 (0.5)	6.1 (0.5)	6.6 (0.5)
	3	90	5.8 (0.6)	5.7 (0.5)	5.8 (0.5)
	4	120	5.7 (0.7)	5.6 (0.6)	5.5 (0.5)
	5	180	4.7 (0.7)	4.6 (0.7)	4.5 (0.6)
Average amount of study product per day (mL/day)	1	30	738.0 (128.9)	743.7 (125.3)	-
	2	60	777.8 (92.1)	778.0 (91.4)	-
	3	90	815.4 (68.0) ^c^	797.3 (64.0)	-
	4	120	858.2 (83.8)	844.9 (56.2)	-
	5	180	842.8 (116.7)	837.7 (121.1)	-
Average energy intake from study product (kcal/day).	1	30	487.1 (85.1)	490.8 (82.7)	-
	2	60	513.3 (60.8)	513.5 (60.4)	-
	3	90	538.2 (44.9) ^c^	526.2 (42.2)	-
	4	120	566.4 (55.3)	557.7 (37.1)	-
	5	180	556.2 (77.1)	552.9 (79.9)	-

^a^ Number of observations in CF: V1, V2: *n* = 157, V3, V4, V5: *n* = 155. ^b^ In CF and eHF, only IP feedings are considered, in BF, only breastfeeding meals are considered. One infant in the CF group received 3.0 breastfeeding meals per day at V1. No infant in the eHF group received any breastfeeding. ^c^ CF vs. eHF *p* = 0.0410 for average amount of study product per day as well as for average energy intake from study product. BF: breastfed reference group; CF: control formula; eHF: formula manufactured from extensively hydrolysed whey protein; FAS: full analysis set; *n*: number of observations; SD: standard deviation; V: visit

**Table 5:** Descriptive statistics of number of adverse events and serious adverse events between enrolment and 180 days of age (V5) (ITT).

	eHF (*n* = 160)		CF (*n* = 158)		BF (*n* = 41)	
	Infants *n* (%)	Events *n*	Infants *n* (%)	Events *n*	Infants *n* (%)	Events *n*
All adverse events	10 (6.3)	10	6 (3.8)	7	0 (0.0)	0
All serious adverse events	0 (0.0)	0	1 (0.6)	1	0 (0.0)	0

BF: breastfed reference group; CF: control formula; eHF: formula manufactured from extensively hydrolysed whey protein; ITT: intention-to-treat population; n: number of observations; V: visit.

## Data Availability

The data that support the findings of this study are available from the corresponding author upon reasonable request.

## Supplementary Materials

The following supporting information can be downloaded at: https://www.mdpi.com/2072-6643/15/8/1901/s1, Table S1: Weight gain (g/day) between 30 days of age (V1) and 120 days of age (V4)—Sensitivity analysis including potential confounding factors. Table S2: Mean daily gain in weight, length and head circumference from 30 days of age (V1) (FAS), mean (SD). Table S3: Feedings characteristics (PPS), mean (SD). Table S4: Descriptive statistics of intervention-emergent adverse events by MedDRA coding (ITT). Figure S1: Observed mean z-scores for BMI-for-age and weight-for-length between birth and 180 days of age (V5) (FAS).
