# Correction: Otten et al. Safety and Suitability of an Infant Formula Manufactured from Extensively Hydrolysed Protein in Healthy Term Infants. *Nutrients* 2023, *15*, 1901

**DOI:** 10.3390/nu15204347

**Published:** 2023-10-12

**Authors:** Lindsey Otten, Elisabeth Schelker, Hanna Petersen, Antonia Nomayo, Manja Fleddermann, Bianca M. Arendt, Theresa Britzl, Elisabeth M. Haberl, Frank Jochum

**Affiliations:** 1 Department of Pediatrics, Evangelisches Waldkrankenhaus Spandau, Stadtrandstr. 555, 13589 Berlin, Germany; lindsey.otten@jsd.de (L.O.); elisabeth.schelker@jsd.de (E.S.); hanna.petersen@jsd.de (H.P.); antonia.nomayo@jsd.de (A.N.); 2 HiPP GmbH & Co. Vertrieb KG, Georg-Hipp-Str. 7, 85276 Pfaffenhofen an der Ilm, Germany; manja.fleddermann@hipp.de (M.F.);; 3 Brandenburg Medical School Theodor Fontane (MHB), Fehrbelliner Str. 38, 16816 Neuruppin, Germany

## Error in Figure

In the original publication [1], Figure 3 in the main text and Figure S1 in the Supplementary Material were incorrect as published. The published figures mistakenly did not include the follow-up period. The corrected Figure 3 and Figure S1 appear below. The authors apologize for any inconvenience caused and state that the scientific conclusions are unaffected. This correction was approved by the Academic Editor. The original publication has also been updated.

**Figure 3:**
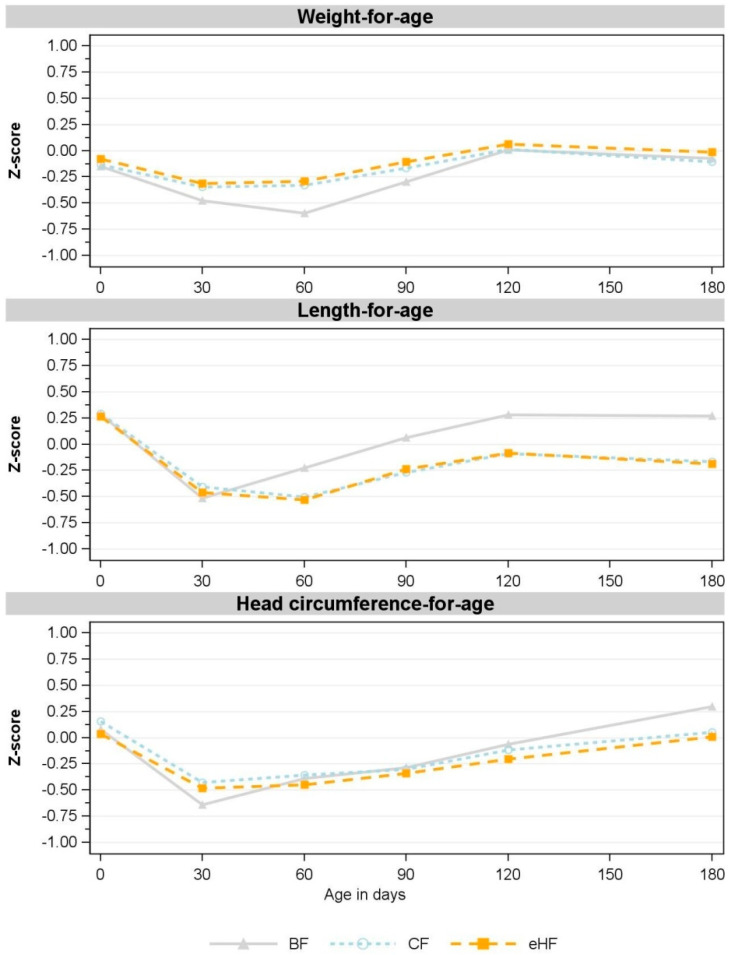
Observed mean for weight-for-age, length-for-age and head circumference-for-age z-scores between birth and 180 days of age (V5) (FAS). V1: 30 days; V2: 60 days; V3: 90 days; V4: 120 days; V5: 180 days. Missing values in CF: weight-for-age: V1: *n* = 1; V3: *n* = 3; V4, V5: *n* = 2; length-for-age: V1, V3: *n* = 1, V4, V5: *n* = 2; head circumference-for-age: V1: *n* = 1, V3, V4, V5: *n* = 2. Missing value in eHF: length-for-age: V4: *n* = 1. Missing value in BF: head circumference-for-age: V3: *n* = 1. BF: breastfed reference group; CF: control formula; eHF: infant formula manufactured from extensively hydrolysed whey protein; FAS: full analysis set; *n*: number of observations; V: visit.

**Figure S1:**
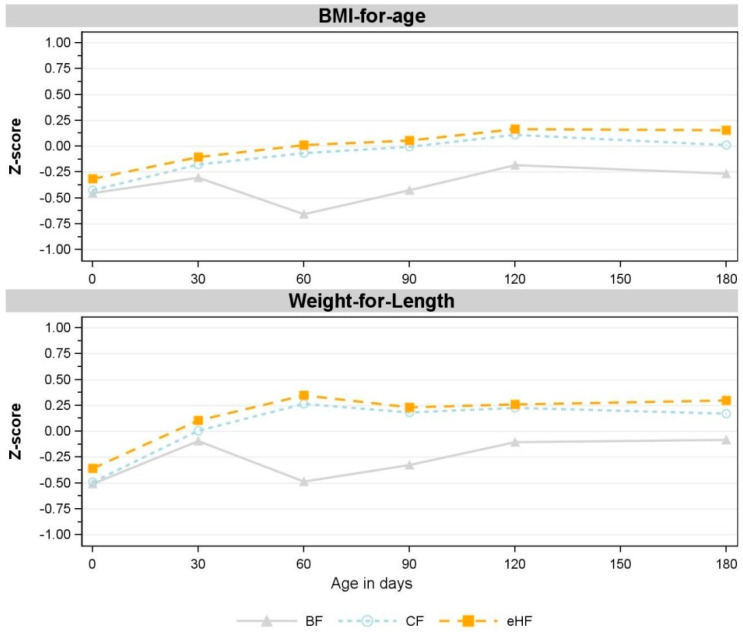
Observed mean z-scores for BMI-for-age and weight-for-length between birth and 180 days of age (V5) (FAS). V1: 30 days; V2: 60 days; V3: 90 days; V4: 120 days; V5: 180 days. No statistically significant differences between CF and eHF were observed. Missing values in CF: BMI-for-age: V1: *n* = 2; V3: *n* = 3; V4, V5: *n* = 2; weight-for-length: V1: *n* = 2; V3: *n* = 3, V4, V5: *n* = 2. Missing values in eHF: BMI-for-age: V4: *n* = 1; weight-for-length: V4: *n* = 1. BF: breastfed reference group; BMI: body mass index (kg/m^2^); CF: control formula; eHF: formula manufactured from extensively hydrolysed whey protein; FAS: full analysis set; *n*: number of observations; V: visit.
