# Supplementary material for: Safety and Suitability of an Infant Formula Manufactured from Extensively Hydrolysed Protein in Healthy Term Infants
Source: Nutrients. 2023 Apr 14;15(8):1901. doi: 10.3390/nu15081901 (PMC10146495; doi:10.3390/nu15081901)
Supplement: Supplementary file 1 [file nutrients-15-01901-s001.zip › nutrients-2317273-supplementary.pdf]

Supplementary Materials (Otten et al. Nutrients 2023)

Table S1: Weight gain (g/day) between 30 days of age (V1) and 120 days of age (V4) – Sensitivity analysis including potential confounding factors

		n	LSM	SEM	95% CI (two-sided)	Two-sided superiority *p* value	97.5% CI (one-sided)	One-sided non-inferiority *p* value[a]
**PPS**	**eHF**	149	28.99	0.68	[27.29;30.68]			
	**CF**	148	28.79	0.69	[27.07;30.51]			
	**eHF vs CF**		0.20	0.48			[-0.75;+inf]	<0.0001
	**Product**					0.6784		
	**Baseline weight**					0.0007		
	**Sex**					0.1470		
	**Birth weight**					0.5449		
	**Gestational age**					0.0040		
	**Age of mother (at infant birth)**					0.0510		
	**BMI of mother (at delivery)**					0.0174		
	**Educational level of mother**					0.6572		
**FAS**	**eHF**	158	28.85	1.09	[26.37;31.33]			
	**CF**	155	28.74	1.09	[26.24;31.23]			
	**eHF vs CF**		0.11	0.49			[-0.85;+inf]	<0.0001
	**Product**					0.8170		
	**Baseline weight**					0.0015		
	**Sex**					0.0262		
	**Birth weight**					0.5287		
	**Gestational age**					0.0332		
	**Age of mother (at infant birth)**					0.2374		
	**BMI of mother (at delivery)**					0.1443		
	**Educational level of mother**					0.7183		

[a] non-inferiority margin: -3 g/day

BMI: body mass index (kg/m^2); CF: control formula; CI: confidence interval; eHF: infant formula manufactured from extensively hydrolysed whey protein; FAS: full analysis set; inf: infinity; LSM: least

square means; *n*: number of observations; PPS: per protocol set; SEM: standard error of mean

Table S2: Mean daily gain in weight, length and head circumference from 30 days of age (V1) (FAS), mean (SD)

	Visit	Age at visit (days)	eHF (*n* = 158)	CF (*n* = 157)[a]	BF (*n* = 41)[b]
**Weight gain (g/day)**	2	60	33.6 (9.1)	33.4 (9.5)	29.4 (4.8)
	3	90	31.5 (6.1)	31.0 (5.7)	30.1 (4.6)
	4	120	29.1 (4.9)	28.8 (4.4)	29.2 (4.1)
	5	180	23.2 (4.1)	22.7 (3.2)	23.1 (3.8)
**Length gain (mm/day)**	2	60	1.1 (0.5)	1.1 (0.5)	1.3 (0.6)
	3	90	1.1 (0.4)	1.1 (0.4)	1.2 (0.5)
	4	120	1.1 (0.3)	1.0 (0.3)	1.1 (0.4)
	5	180	0.9 (0.2)	0.9 (0.2)	0.9 (0.3)
**Head circumference gain (mm/day)**	2	60	0.6 (0.3)	0.6 (0.3)	0.7 (0.3)
	3	90	0.5 (0.2)	0.5 (0.2)	0.6 (0.2)
	4	120	0.5 (0.1)	0.5 (0.2)	0.5 (0.1)
	5	180	0.4 (0.1)	0.4 (0.1)	0.5 (0.1)

No statistically significant differences between CF and eHF were observed.

[a] Missing values in CF: weight gain: V2: *n* = 1; V3: *n* = 3; V4, V5: *n* = 2 length gain: V2: *n* = 1; V3: *n* = 2; V4, V5: *n* = 3; head circumference gain: V2: *n* = 1; V3, V4, V5: *n* = 3

[b] Missing values in BF: head circumference gain: V3: *n* = 1

BF: breastfed reference group; CF: control formula; eHF: infant formula manufactured from extensively hydrolysed whey protein; FAS: full analysis set; *n*: number of observations; SD: standard deviation; V: visit

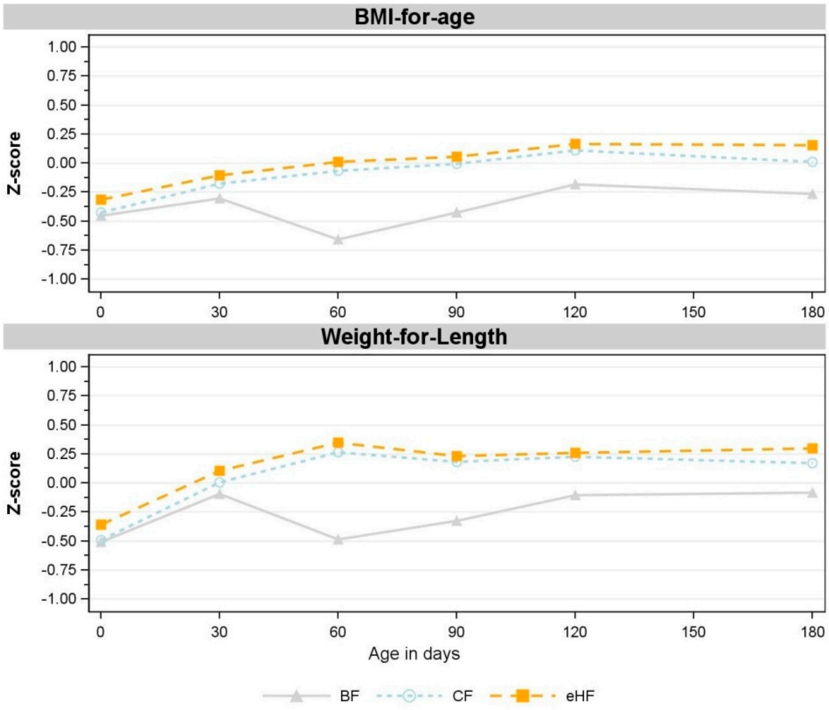

Figure S1*:* Observed mean z-scores for BMI-for-age and weight-for-length between birth and 180 days of age (V5) (FAS)

V1: 30 days; V2: 60 days; V3: 90 days; V4: 120 days; V5: 180 days

No statistically significant differences between CF and eHF were observed.

Missing values in CF: BMI-for-age: V1: *n* = 2; V3: *n* = 3; V4, V5: *n* = 2; weight-for-length: V1: *n* = 2; V3: *n* = 3, V4, V5: *n* = 2

Missing values in eHF: BMI-for-age: V4: *n* = 1; weight-for-length: V4: *n* = 1

BF: breastfed reference group; BMI: body mass index (kg/m^2); CF: control formula; eHF: formula manufactured from extensively hydrolysed whey protein; FAS: full analysis set; *n*: number of observations; V: visit

Table S3: Feedings characteristics (PPS), mean (SD)

	Visit	Age at visit (days)	eHF (*n* = 149)	CF (*n* = 148)	BF (*n* = 41)
**Average number of feedings per day**	1	30	7.1 (0.8)	7.0 (0.8)	7.4 (0.6)
	2	60	6.1 (0.5)	6.1 (0.5)	6.6 (0.5)
	3	90	5.8 (0.5)	5.7 (0.5)	5.8 (0.5)
	4	120	5.7 (0.6)	5.6 (0.6)	5.5 (0.5)
	5	180	4.7 (0.6)	4.6 (0.6)	4.5 (0.6)
**Average amount of study product per day (mL/day)**	1	30	741.9 (125.5)	745.6 (125.0)	-
	2	60	777.9 (90.2)	777.0 (91.3)	-
	3	90	813.1 (57.7)[a]	794.4 (62.0)	-
	4	120	852.7 (64.2)	844.2 (56.9)	-
	5	180	844.4 (110.4)	838.0 (112.2)	-
**Average energy intake from study product (kcal/day)**	1	30	489.7 (82.8)	492.1 (82.5)	-
	2	60	513.4 (59.5)	512.8 (60.2)	-
	3	90	536.7 (38.1)[a]	524.3 (40.9)	-
	4	120	562.8 (42.4)	557.2 (37.6)	-
	5	180	557.3 (72.9)	553.1 (74.1)	-

[a] CF vs. eHF $p = 0.0218$ for average amount of study product per day as well as for average energy intake from study product.

BF: breastfed reference group; CF: control formula; eHF: formula manufactured from extensively hydrolysed whey protein; *n*: number of observations; PPS: per protocol set; SD: standard deviation; V: visit

Table S4: Descriptive statistics of intervention-emergent adverse events by MedDRA coding (ITT)

	eHF (*n* = 160)		CF (*n* = 158)		BF (*n* = 41)	
	**Infants** ***n*** **(%)**	**Events** ***n***	**Infants** ***n*** **(%)**	**Events** ***n***	**Infants** ***n*** **(%)**	**Events** ***n***
**All intervention-emergent adverse events**	10 (6.3%)	14	6 (3.8%)	11	0 (0.0%)	0
**System organ class (SOC)**						
Gastrointestinal disorders	5 (3.1%)	5	3 (1.9%)	3	0 (0.0%)	0
General disorders and administration site conditions	1 (0.6%)	1	1 (0.6%)	2	0 (0.0%)	0
Infections and infestations	2 (1.3%)	2	2 (1.3%)	2	0 (0.0%)	0
Investigations	1 (0.6%)	1	0 (0.0%)	0	0 (0.0%)	0
Psychiatric disorders	2 (1.3%)	2	2 (1.3%)	2	0 (0.0%)	0
Respiratory, thoracic and mediastinal disorders	2 (1.3%)	2	0 (0.0%)	0	0 (0.0%)	0
Skin and subcutaneous tissue disorders	0 (0.0%)	0	1 (0.6%)	1	0 (0.0%)	0
Surgical and medical procedures	1 (0.6%)	1	1 (0.6%)	1	0 (0.0%)	0
**Preferred term (PT)**						
Abdominal pain	1 (0.6%)	1	1 (0.6%)	1	0 (0.0%)	0
Agitation	1 (0.6%)	1	2 (1.3%)	2	0 (0.0%)	0
Body temperature increased	1 (0.6%)	1	0 (0.0%)	0	0 (0.0%)	0
Bronchitis	1 (0.6%)	1	0 (0.0%)	0	0 (0.0%)	0
Constipation	2 (1.3%)	2	1 (0.6%)	1	0 (0.0%)	0
Crying	1 (0.6%)	1	1 (0.6%)	1	0 (0.0%)	0
Fungal infection	0 (0.0%)	0	1 (0.6%)	1	0 (0.0%)	0
Immunisation	1 (0.6%)	1	1 (0.6%)	1	0 (0.0%)	0
Pyrexia	0 (0.0%)	0	1 (0.6%)	1	0 (0.0%)	0
Rash	0 (0.0%)	0	1 (0.6%)	1	0 (0.0%)	0
Regurgitation	0 (0.0%)	0	1 (0.6%)	1	0 (0.0%)	0
Respiratory tract infection	1 (0.6%)	1	0 (0.0%)	0	0 (0.0%)	0
Restlessness	1 (0.6%)	1	0 (0.0%)	0	0 (0.0%)	0
Rhinitis	1 (0.6%)	1	1 (0.6%)	1	0 (0.0%)	0
Rhinorrhoea	1 (0.6%)	1	0 (0.0%)	0	0 (0.0%)	0
Vomiting	2 (1.3%)	2	0 (0.0%)	0	0 (0.0%)	0

When applying MedDRA coding, one AE can be allocated to multiple SOCs/PTs. Therefore, the total number of MedDRA-coded AEs exceeds the total number of recorded AEs.

AE: adverse event; BF: breastfed reference group; CF: control formula; eHF: infant formula manufactured from extensively hydrolysed whey protein; ITT: intention-to-treat population; MedDRA: Medical Dictionary for Regulatory Activities; *n*: number of observations/events; PT: preferred term; SOC: system organ class
